# Towards Prenatal Biomonitoring in North Carolina: Assessing Arsenic, Cadmium, Mercury, and Lead Levels in Pregnant Women

**DOI:** 10.1371/journal.pone.0031354

**Published:** 2012-03-09

**Authors:** Alison P. Sanders, Kaye Flood, Shu Chiang, Amy H. Herring, Leslie Wolf, Rebecca C. Fry

**Affiliations:** 1 Department of Environmental Sciences and Engineering, Gillings School of Global Public Health, University of North Carolina, Chapel Hill, North Carolina, United States of America; 2 State Laboratory of Public Health, North Carolina Department of Health and Human Services, Raleigh, North Carolina, United States of America; 3 Department of Biostatistics, Gillings School of Global Public Health, and Carolina Population Center, University of North Carolina, Chapel Hill, North Carolina, United States of America; Mental Health Research Institute of Victoria, Australia

## Abstract

Exposure to toxic metals during the prenatal period carries the potential for adverse developmental effects to the fetus, yet such exposure remains largely unmonitored in the United States. The aim of this study was to assess maternal exposure to four toxic metals (arsenic (As), cadmium (Cd), mercury (Hg), and lead (Pb)) in a cohort of pregnant women in North Carolina. We analyzed blood samples submitted to the North Carolina Department of Health and Human Services for blood typing to assess toxic metal levels in pregnant women (n = 211) across six North Carolina counties. Whole blood metal concentrations were measured by inductively coupled plasma mass spectrometry. The association between maternal characteristics, including county of residence, age, and race, and metal exposure was analyzed using multiple linear regression analysis. A large fraction of the blood samples showed detectable levels for each of the four metals. Specifically, As (65.7%), Cd (57.3%), Hg (63.8%), and Pb (100%) were detected in blood samples. Moreover, compared with adult females participating in the *Fourth National Report on Human Exposure to Environmental Chemicals* and guidelines for pregnant women, some women in the sample population exceeded benchmark levels of Cd, Hg, and Pb. Evidence from this pilot study indicates that pregnant women in North Carolina are exposed to As, Cd, Hg, and Pb and suggests that factors related to maternal county of residence and race may impact maternal exposure levels. As increased levels of one or more of these metals *in utero* have been associated with detrimental developmental and reproductive outcomes, further study is clearly warranted to establish the impacts to newborns.

## Introduction

Arsenic (As), cadmium (Cd), mercury (Hg), and lead (Pb) are metals ranked among the top ten most toxic substances by the Agency for Toxic Substances and Disease Registry [1]. Maternal exposures to toxic metals may result from diet, air, drinking water, occupational exposures, and/or tobacco use. Evidence suggests that each of these metals is able to cross the placental barrier resulting in prenatal exposure [2], [3], [4], [5] and maternal blood levels of these contaminants correlate with umbilical cord blood levels [5], [6]. Biologically, prenatal exposure to metals is of concern for a variety of reasons. For example, prenatal exposure to arsenic is associated with later life health effects in adults [7], [8], [9] including increased mortality and increased risk of lung and liver cancer [10], [11], [12]. In addition, *in utero* exposure to arsenic has been shown to alter genomic signaling of key biological pathways [13]. Furthermore, maternal exposures to toxic metals can increase the risk for poor birth outcomes, including low birth weight [3], [14], [15], reduced fetal growth [16], and reproductive and cognitive deficits in adolescents [17], [18], [19].

Toxic metal exposures *in utero* and during childhood may result in significant health effects to individuals that manifest cumulatively to the detriment of populations. U.S. annual economic losses due to decreased productivity from environmental exposure to Hg and Pb were estimated as $8.7 and $43.4 billion respectively [20], [21]. In spite of strong evidence to indicate the adverse health effects of metals to susceptible populations such as newborns, few statewide biomonitoring programs are in place. For example, while North Carolina has established a successful program for childhood lead screening [22], there remains no prenatal assessment of environmental contaminants. This is of concern in North Carolina because the occurrence of metals including As, Pb, and Hg has been reported in environmental reservoirs with the potential for human exposure [23], [24], [25].

Known health risks are associated with exposure to heavy metals, particularly during the periconceptional and prenatal periods. Established recommended advisory blood levels of 5.8 µg/L Hg and 10.0 µg/dL Pb are considered protective of human health for the general population [26], [27]. However, because cord blood levels of Hg and Pb have been found to exceed maternal blood levels, the maternal blood levels of 3.5 µg/L Hg and 5.0 µg/dL Pb are recommended for the protection of fetal health [26], [28], [29]. Blood levels in individuals exceeding these reference concentrations are associated with decreased IQ and cognitive function [20], [26]. Despite evidence of associated detrimental health effects, there are currently no biological advisory levels pertaining to As or Cd for pregnant women or the general population.

The major aim of this pilot study was to assess blood levels of As, Cd, Hg, and Pb in pregnant women residing in six North Carolina counties and to examine associations of metal levels according to maternal age, race, and county of residence. Evidence gathered in this study will be used to further assess maternal and prenatal exposures in future studies and to provide impetus for targeted biomonitoring programs or public health campaigns in North Carolina. Biomonitoring of maternal levels of toxic metals in targeted populations may help reduce prenatal exposures and subsequently the potential for future adverse developmental effects.

## Methods

### Study Design

To assess blood levels of toxic metals in pregnant women, we analyzed blood samples submitted to the North Carolina Department of Health and Human Services (NCDHHS) State Laboratory of Public Health for blood group typing. Residual volumes of blood originally submitted for group typing were analyzed for As, Cd, Hg, and Pb. Data were provided under the context of a data use agreement for a limited data set using a data custodian as an intermediary to protect identifiable information. This study was reviewed by the UNC Public Health-Nursing IRB (#09-1399) and was determined to be exempt from review according to regulatory category 4 under the National Institutes of Health description on Research on Human Specimens (44 CFR 46.101(b)). Similar strategies that assess metal levels in samples collected under the auspices of existing public health monitoring programs have been used to establish environmental contaminant levels in individuals [30].

Women in this study were North Carolina residents in their third trimester of pregnancy and receiving prenatal care at local health departments. The mothers' venous blood samples were collected between October 2009 and January 2011. Bladen, Cumberland, Richmond, Stanly, Union, and Wake Counties were selected for analysis based on trends in private well water metal levels reported in publicly available data [31]. Throughout the remainder of the manuscript county identities are coded and designated as A–F, using random assignment. Data collected included: (1) pregnant women's (n = 211) whole blood level of As, Cd, Hg, and Pb, (2) county of residence, (3) age, and (4) race. No additional demographic information on subjects was requested in accordance with the limited data use agreement.

### Blood Analysis

The North Carolina Department of Health and Human Services (NCDHHS) for blood typing group is certified by the U.S. DHHS as compliant with the 1988 Clinical Laboratory Improvement Amendment (CLIA). In accordance with CLIA certification, the laboratory adheres to quality assessment/quality control (QA/QC) requirements with oversight by the Centers for Medicare and Medicaid Services. Blood samples from non-fasting pregnant women were collected into phlebotomy tubes containing ethylenediaminetetraacetic acid (EDTA) as an anticoagulant and stored at 4°C prior to processing at the Division of Public Health State Laboratory. Whole blood analysis was performed via Inductively Coupled Plasma Dynamic Reaction Cell Mass Spectrometry (ICP/DRC/MS) according to the US CDC laboratory methods for metals in whole blood, DLS Method code CTL-TMS 3.01 [32], [33]. The samples were analyzed on a Perkin Elmer Elan DRC II ICP/MS instrument. Arsenic was measured in blood using the DRC mode to remove isobaric interference from Argon Chloride at mass 75. Iridium was used as an internal standard for all elements. The limits of detection (LODs) were 0.23 µg-As/L, 0.11 µg-Cd/L, 0.23 µg-Hg/L, and 0.13 µg-Pb/dL. The results represent levels of total metal concentrations; speciation of metals was not performed. The ICP-MS analyses for Cd, Hg, and Pb were similar to those used in the Fourth National Report on Human Exposure to Environmental Chemicals (NHANES IV).

### Statistical analysis

Statistical analysis was conducted with the statistical package SAS 9.2 (SAS Institute Inc., Cary, North Carolina). Blood samples with metal levels below the LOD were assigned an imputed value equal to LOD/√2, as in NHANES IV [34]. The data were log-transformed to address the non-normal distribution of metal concentrations. Spearman's correlation coefficient estimates among metal pairs and corresponding p-values were calculated. Linear regression was performed on every pairwise metal combination by adjusting for maternal age to test for associations between blood metal levels and race (NHW served as the referent group). Additional multivariate analyses were performed adjusting for maternal age, race, and categorized county of residence to assess the relationship between metal levels and residence in individual counties. Furthermore, differences between the mean metal levels of individual counties were compared using Scheffe's test (α = 0.05).

## Results

Here we monitored metal levels in blood samples collected from pregnant women (n = 211) residing in North Carolina between October 2009 and January 2011. Study participant characteristics are presented in Table 1. The average woman's age was 25 years and ranged from 15 to 43 years. The study population was 2% Asian, 38% non-Hispanic Black (NHB), 6% Hispanic, and 55% non-Hispanic White (NHW). The monitored women were residents of County A (n = 50), County B (n = 28), County C (n = 50), County D (n = 13), County E (n = 25), and County F (n = 45) in North Carolina.

**Table 1:** Characteristics of the pregnant women (n = 211) in the third trimester of pregnancy.

**Race/ethnicity, n (%)**	
Asian	4 (2%)
Non-Hispanic Black	79 (38%)
Hispanic	13 (6%)
Non-Hispanic White	115 (55%)
**County of residence, n (%)**	
County A	50 (24%)
County B	28 (13%)
County C	50 (24%)
County D	13 (6%)
County E	25 (12%)
County F	45 (22%)

**Figure 1:**
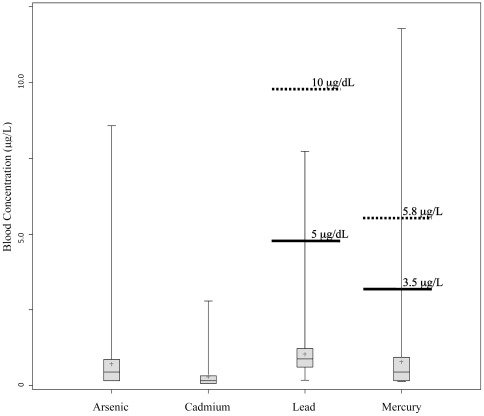
Boxplots of blood levels of As, Cd, Pb, and Hg in 211 pregnant women residing in North Carolina. Solid horizontal lines indicate guideline values believed to be protective of fetal health. Dashed lines represent established guideline levels for the general public. No values have been established for As or Cd. The center horizontal line in each box blot corresponds to the sample median and the central plus sign corresponds to the sample mean. Horizontal lines also represent the 25^th^ and 75^th^ percentiles as well as minimum and maximum measured values. *Lead units are expressed in µg/dL.

From the 211 individuals in the sample population, blood metal levels were detected among 138 for As (65.7%), 121 for Cd (57.3%), 134 for Hg (63.8%), and 211 for Pb (100%) samples. Descriptive statistics of metal levels are presented in Table 2. The 95^th^ percentile whole blood Cd, Hg, and Pb levels among women of childbearing age (age 16–49) participating in NHANES IV (n = 4,241) were reported as 1.60 µg/L, 4.40 µg/L, and 3.50 µg/dL, respectively [34]. A total of three (1.4%), five (2.4%), and four (1.9%) women in the study population exceeded these levels for Cd, Hg and Pb, respectively. Arsenic in whole blood was not measured in NHANES IV and therefore could not be compared to these data. However, three (1.4%) women here had levels of As comparable to those reported for an environmentally exposed cohort of pregnant women [2]. Blood Cd levels were within previously reported ranges for pregnant women, however, some Hg and Pb levels exceeded reported levels [35]. Specifically, of the pregnant women sampled, four women (1.9%) had Hg concentrations above the EPA blood guideline of 5.8 µg/L and two women (0.94%) had Pb levels above 5.0 µg/dL (the CDC blood lead advisory level for pregnant women) [26], [27] (Figure 1). Additionally, five women (2.3%) had blood Hg levels above the 3.5 µg/L level of concern during pregnancy [28], [29].

**Table 2:** Detectable levels and geometric averages of the four toxic metals in women.

Blood Metals	n	% Detected	Geometric Mean^a^ (range)
Arsenic	210	65.7	0.445 (<0.23–8.58) µg/L
Cadmium	211	57.3	0.181 (<0.11–2.79) µg/L
Mercury	210	63.8	0.453 (<0.23–11.78) µg/L
Lead	211	100	0.890 (0.19–7.72) µg/dL

a The geometric mean was calculated with measures <LOD assigned values of LOD/√2 [35].

Our analysis revealed relationships among some sample population characteristics and maternal metal levels. Table 3 shows the unadjusted Spearman correlation coefficients computed for each pairwise combination of metals. Notably, maternal levels of As and Hg positively covaried in the study population (r = 0.45, p<0.001). This suggests that for some pregnant mothers, As and Hg are elevated in tandem. Specifically samples collected from Counties A, C, and F demonstrated a significant positive correlation between maternal As and Hg (p<0.05, data not shown). No other metal pair correlations were statistically significant at the α = 0.001 significance level.

**Table 3:** Spearman's rank correlation coefficients for metals in blood of North Carolina pregnant women (n = 211).

	As	Cd	Hg	Pb
As	1.	−0.14*	0.45**	0.12
Cd		1	−0.21*	0.07
Hg			1	0.19*
Pb				1

* P<0.05;

** P<0.001;

Data below detect were assigned imputed values of LOD/√2.

Our data also indicate that maternal race and residence in certain counties were associated with elevated body burden of some toxic metals. Linear regression of maternal race on metal levels adjusted for maternal age is presented in Table 4. The results reveal that NHB maternal race was associated with increased blood As (p<0.05) and Cd levels (p<0.001) when compared to NHW mothers. Asian women in this study also appear to have elevated levels of As, Cd, and Hg, although the sample size was small and additional study is needed to verify this trend.

**Table 4:** Linear regression of age-adjusted maternal race on blood metal levels (Beta coefficient and 95% CI).

	Asian	Hispanic	NHB	NHW (ref)
As	0.51 (0.14–0.88)*	−0.13 (−0.35–0.08)	0.12 (0.02–0.23)*	–
Cd	0.39 (0.01–0.77)*	0.20 (−0.02–0.41)	0.25 (0.15–0.36)**	–
Hg	0.64 (0.23–1.05)*	−0.07 (−0.31–0.16)	0.01 (−0.11–0.12)	–
Pb	−0.01 (−0.24–0.22)	−0.08 (−0.21–0.05)	−0.07 (−0.13–0.00)*	–

NHW served as the referent group.

* p<0.05;

** P<0.001;

Metal levels were log-transformed.

Table 5 shows the geometric mean metal levels by coded maternal county of residence. The compared differences among average metal levels in individual counties using Scheffe's test after adjusting for maternal age and race are also displayed. The highest geometric mean As level (0.87 µg/L) was in County F. The As levels in samples collected from Counties C and F were significantly elevated when compared to at least one other county (Scheffe's p<0.05; Table 5). Cd levels in samples collected from Counties A and C were significantly elevated when compared to at least one other county (Scheffe's p<0.05), and the highest geometric mean level of Cd (0.27 µg/L) was in County A. Among Hg levels, samples from County F had the highest geometric mean Hg (0.87 µg/L), and average levels in Counties D and F were significantly elevated when compared to one or more other counties (Scheffe's p<0.05; Table 5). Finally, maternal blood levels of Pb in County C had the highest geometric mean (1.28 µg/L), and were significantly elevated compared individually to those from Counties A, E, and F (Scheffe's p<0.05). Notably, samples collected from County C had the highest levels of As, Cd, and Pb. Samples collected from County F had elevated average levels of As and Hg. Counties A and D had elevated average levels of Hg and Cd, respectively. Average Pb and Hg levels exceeded those reported for women participating in NHANES IV in County C and County F, respectively (Table 5) [34].

**Table 5:** Concentration of As, Cd, Hg, and Pb in maternal blood collected from women residing in selected North Carolina counties – reported as the geometric mean.

	Maternal blood concentration^a^ (µg/L)*			
	As	Cd	Hg	Pb
County A (n = 50)	0.29	0.27^B^	0.28	0.70
County B (n = 28)	0.36	0.14	0.39	0.89
County C (n = 50)	0.49^A^	0.23^B^	0.40	1.28^D^
County D (n = 13)	0.33	0.09	0.70^C^	1.02
County E (n = 25)	0.39	0.11	0.45	0.69
County F (n = 45)	0.87^A^	0.17	0.87^C^	0.86
Total (n = 211)	0.44	0.18	0.45	0.89
NHANES IV	NA	0.33	0.78	1.22

Abbreviations: NA not available; NHANES: Geometric mean levels (2003–2004) reported for women (n = 4,241) participating in NHANES IV [35].

a Concentration reported as the geometric mean blood levels collected from women in the third trimester of pregnancy.

* Units of Pb are expressed in µg/dL.

A Arsenic levels in County C were significantly elevated compared individually to County A (Scheffe's p<0.05). As levels in County F were significantly elevated compared to each of the other five counties individually (Scheffe's p<0.05).

B Cadmium levels in County A were significantly elevated compared individually to County B, D, and E (Scheffe's p<0.05). Cd levels in County C were significantly elevated compared individually to County D and E (Scheffe's p<0.05).

C Mercury levels in County F were significantly elevated compared individually to County A, B, and C (Scheffe's p<0.05). Hg levels in County D were significantly elevated compared individually to County A (Scheffe's p<0.05).

D Lead levels in County C were significantly elevated compared individually to County A, E, and F (Scheffe's p<0.05).

## Discussion

### Pregnant women are exposed to toxic metals

The aim of this study was to assess toxic metal exposure in pregnant mothers in North Carolina. The data suggest that indeed pregnant women in North Carolina are exposed to toxic metals. For each of the four toxic metals studied, more than 50% of the women had detectable blood levels. Specifically, depending on the metal detectable blood levels were found in 57–100% of the samples. Most of the metal measurements revealed levels of exposure that did not fall outside regulated limits. For some individuals, however, blood levels of toxic metals exceeded the 95^th^ percentile of Cd, Hg, and Pb reported in the U.S. population. Moreover, a few women had metal levels above current guideline levels adopted for health protection. Specifically, some women had blood metal levels above reference values indicative of a threshold for adverse health effects: five women (2.3%) had blood Hg levels above 3.5 µg/L and two women (0.94%) had Pb levels greater than 5.0 µg/dL (the CDC blood lead advisory). Since no current threshold levels exist for As in blood we compared our findings with a previous study by Concha et al [2]. Three women in our study showed levels similar to those reported among As-exposed pregnant women served by contaminated public water supplies containing up to 200 µg/L As (blood range: 5.6–13 µg/L) [2]. In addition, thirty-seven women had blood levels greater than 1 µg/L, a level previously associated with known exposure to As in drinking water [36].

### As and Hg are positively correlated in pregnant mothers' blood

Environmental exposures in the general public more commonly occur as mixtures than as single-contaminant exposures. Contaminant mixtures can contribute to an individuals' susceptibility and potentially have synergistic effects resulting in disease manifestation [37], [38]. Here we examined the correlation of the four toxic metals among the pregnant women. Notably, we report a relationship between maternal As and Hg blood levels. An implication of this finding is that maternal and fetal body burden may result from combined or interactive effects of more than one metal. Thus, concomitant exposure is an important consideration in future studies that assess *in utero* health outcomes from metal exposure.

### Non-Hispanic Black maternal race is associated with increased Cd

The findings also highlight associations between blood metal levels and maternal race. Our data suggest that across the sample population NHB maternal race was associated with increased Cd exposure compared to NHW women. To our knowledge this association has not been previously reported. Poor birth outcomes in North Carolina and across the U.S. disproportionately affect infants born to NHB mothers [39], [40], despite the lack of a definitive causal mechanism. Our findings suggest that the role of maternal race in birth outcome etiology may be confounded by metal exposure, and further investigation of social, environmental, and genetic factors disproportionately affecting NHB mothers is needed.

### County of residence is associated with mothers' metal levels

This initial study suggests that on average, women residing in various North Carolina counties experience differential exposure to toxic metals during pregnancy. We show that on average, pregnant women residing in Counties C and F were likely to have higher levels of As when compared to women residing in the other counties. Exposure to As primarily occurs through ingestion of food and drinking water [41]. In public drinking water supplies, allowable levels of As can range up to the EPA Maximum Contaminant Level (MCL) of 10 µg/L. As such, individuals can be exposed to detectable levels of As that are still within regulatory guidelines. We have shown recently that in North Carolina, some counties have elevated levels of As in unregulated private well water [23]. Here we show that women living in Counties C and F have elevated blood As, two counties that were not identified as particular outliers in the well water analysis. This could suggest an alternate route of exposure among residents, perhaps through diet. In addition, this study did not select for individuals based on their type of drinking water source. Taken together, these results suggest that a future study of women supplied by private drinking water sources may be prudent.

We also found that on average, pregnant women residing in Counties D and F also had elevated levels of blood Hg. Most Hg exposures in the U.S. are attributable to diet, particularly fish consumption [42]; however, dental amalgams [4] and some industrial processes also contribute to human exposure [43]. In North Carolina, a statewide freshwater fish advisory for mercury (specifically in largemouth bass) has been in effect since 2009. A recent study in Durham County, North Carolina found that the type of fish consumption and corresponding Hg content varied among different demographic groups of pregnant women [24]. There are no reports of public or private well drinking water Hg levels above acceptable EPA MCL standard of 2 µg/L in either of these counties.

Cadmium exposure commonly occurs through food consumption or inhalation of cigarette smoke, although minor exposure can occur from byproducts of industrial processes [44]. Previous studies have shown Cd levels in maternal and cord blood are higher in smokers [45], and that Cd levels among smokers were reportedly 4–5 times higher than nonsmokers [46]. Moreover, Cd levels in pregnant women exposed to second–hand smoke were double that of nonsmokers [6]. In our study, residence in County A was associated with increased maternal blood Cd levels; yet, the smoking rate in this county is lower than the statewide rate (20.9% of adults) [47]. Detectable levels of Cd in public drinking water can range up to 5.0 µg/L (the EPA MCL) and may potentially contribute to low-level exposure in the general population. Further research is needed to establish the source of maternal Cd exposure.

Exposure to Pb can result from various environmental sources such as contaminated dust or soil, drinking water, lead-based paint, cigarette smoke, as well as byproducts of industrial processes [48]. Elevated maternal blood levels of Pb were associated with residence in County C. Similar to the other metals, Pb levels in public drinking water supplies can range up to the 15 µg/L and up to 10% of samples can exceed this level under EPA guidelines. Currently, no evidence indicates that North Carolina public water sources contain endemic levels of lead. While drinking water is a potential route of lead exposure, rural counties may experience additional risk factors for Pb exposure such as older housing and low socioeconomic status that may put mothers at risk [49].

Although the design of this study did not allow us to ascertain exposure sources, both anthropogenic and environmental sources can contribute to an individuals' toxic metal exposure. Body weight, race, occupational factors, duration of residence, socioeconomic status, smoking status, gestational time during pregnancy, and genetic factors can affect the body burden of metals. It is likely that multiple exposure routes and sources may have contributed to the metal levels assessed in this study and warrant future follow-up.

### Biomonitoring programs may reduce the risk of prenatal metal exposure

Despite evidence that adverse health effects are attributable to *in utero* metal exposure, currently Minnesota, New York City, and New York State are the only jurisdictions that have active guidelines for monitoring maternal blood lead levels in the U.S. [26]. The initiation of statewide metal biomonitoring programs in the U.S. may be accomplished through collaborations with state programs to better protect public health, particularly in susceptible populations such as pregnant women and children. This study demonstrates that detectable metal levels were present in maternal blood and suggests that further biomonitoring of blood levels of toxic metals in susceptible North Carolina populations is prudent. Furthermore, *in lieu* of existing targeted biomonitoring programs, public health strategies to prevent exposure such as raising awareness among pregnant mothers may be vital to protecting the health of newborns.

In conclusion, this study demonstrates that the pregnant women under study were exposed to four toxic metals at detectable levels. Specifically, we show evidence of elevated levels of As, Hg, and Pb among some women in the third trimester of pregnancy. In addition, we identified statistically significant relationships between maternal As and Hg in blood levels. Furthermore, we report associations between maternal blood metal levels and maternal race as well as county of residence. The increased levels of one or more of these metals are associated with detrimental neurological and physiological developmental outcomes. Informing pregnant women of the sources of toxic metals such as diet (including drinking water), cigarette smoke, and other environmental sources may help to reduce exposures. Targeted national, statewide, and community level programs promoting mothers' awareness of metals exposure and *in utero* effects would each help to protect human health and lessen the economic burden of metal exposure. Biomonitoring programs in North Carolina and other at-risk areas can help to protect the health of expectant mothers and their children.
